# Production of bioactive compounds with bactericidal and antioxidant potential by endophytic fungus *Alternaria alternata* AE1 isolated from *Azadirachta indica* A. Juss.

**DOI:** 10.1371/journal.pone.0214744

**Published:** 2019-04-04

**Authors:** Sohini Chatterjee, Ranjan Ghosh, Narayan Chandra Mandal

**Affiliations:** Mycology and Plant Pathology Laboratory, Department of Botany, Visva-Bharati, Santiniketan, West Bengal, India; Universite Paris-Sud, FRANCE

## Abstract

For combating multidrug-resistant microorganisms, exploration of natural compounds from plant endophytes increases the chance of finding novel compounds. An efficient bioactive metabolites producing endophytic fungal strain AE1 was isolated from leaves of *Azadirachta indica* A. Juss. The metabolites were found to be thermostable, non-proteinacious and produced prominent zones of inhibition against numbers of Gram positive and Gram negative bacteria. Based on 28S rDNA (D1/D2) sequence homology the isolate AE1 was identified as *Alternaria alternata*. Malt extract broth was found effective for the maximum production of bioactive metabolites by the isolate and was subjected for solvent extraction. The Ethyl acetate (EA) fraction of AE1 showed MIC values of 300–400 μg/ml against Gram positive and Gram negative bacteria tested. The cidal mode of action of EA fraction was detected by treating bacterial cultures at mid log phase. Scanning electron microscopic study supported morphological disintegration of bacterial cells. Release of nucleic acid, protein and potassium ions (K^+^) also suggested lysis of bacterial cells or leakage of cell membrane upon treatment. In addition, reduction of the activity of EMP pathway, TCA cycle and gluconeogenic enzymes in all bacteria suggested the interference of antibacterial principles with central carbohydrate metabolic pathways. Thin layer chromatographic separation followed by GC-MS analysis of EA fraction suggested numbers of antimicrobial compound production by AE1. In addition, DPPH free radical as well as superoxide radical scavenging assay also suggested strong antioxidant potential of AE1 with an IC_50_ value of 38.0±1.7 μg/ml and 11.38±1.2 μg/ml respectively. On the basis of above facts it can be concluded that the strain AE1 will be a good source of bioactive compounds having medicinal importance.

## Introduction

Almost all the plants produce a wide array of secondary metabolites for their own benefits, some of which have tremendous biological activities. Since antiquity, plants as a source of medicinal compounds have continued to play a dominant role in maintenance of human health. It has been observed that the bioactive compounds derived from plants in many instances are not actually their own metabolic products rather these are produced by some microorganisms live inside the tissue in a symbiotic fashion which are known as endophytes [1]. Different groups of organisms like fungi, bacteria, mycoplasma and actinomycetes are established as endophytes of plants [2]. For protecting the host plant from different biotic and abiotic stress conditions, they produce different bioactive compounds and in turn they take nutrition from the plants [3,4]. Production of the popular anticancer drug taxol has been reported by an endophytic fungus *Taxomyces andreanae* which grows symbiotically in the bark of the plant *Taxus brevifolia* [5]. This discovery primarily saved a number of *Taxus* plants from being extinction. This is analogically true for many other plants [6,7]. Research is going on in full swing to find out the actual role of both endophytes and plants to produce the bioactive compounds. It is found that the ability for producing bioactive compounds by endophytes is enormous [1].

*Azadirachta indica* A. Juss. (Family- Meliaceae) commonly known as neem is an important medicinal plant. In traditional medicine it is used as a potent therapeutic agent [8]. *A*. *indica* contains a number of bioactive compounds of which azadirachtin, nimbin, nimbidol, nimbidin, sodium nimbinate, salannin and quercetin are important [9]. Verma et al. [10] have identified six endophytic fungal isolates with antimicrobial potential viz.,*Pestalotiopsis* sp., *Alternaria* sp., *Scytalidium* sp., *Colletotrichum* sp. and *Nigrospora* sp. from *Azadirachta indica* A. Juss. grown in Varanasi, India. The diversity of endophytic microorganisms varies from plant to plant, and even in the same plant grown in different habitat [11]. Variation in the endophytic fungal diversity may be related with location, climate and plant age [12,13]. In the present endeavor, we have tried to find out the potent endophytic fungus having biological activities from the leaves of *A*. *indica* grown in Santiniketan, a place located in the lateritic belt of West Bengal, India. The mechanisms of action of antibacterial metabolites produced by the isolate on pathogenic bacteria were deciphered. Antibacterial metabolites were partially purified and identified by GC-MS analysis. Antioxidant potential of the isolate was also checked by DPPH and superoxide free radical scavenging assy.

## Materials and methods

### Collection of plant materials

Healthy and mature leaves of *Azadirachta indica* were randomly collected from Santiniketan (23.6816660°N, 87.6714825°E), West Bengal, India using sterilized polythene bags and brought to the laboratory using an icebox. The leaves were collected from the plants grown abundantly in the medicinal plant garden of Department of Botany, Visva-Bharati, Santiniketan and the permission was taken from the Head of the Department. According to eflora of India published by Botanical Survey of India (BSI), *A*. *indica* A. Juss. is considered as a common plant throughout the country and is also cultivated widely (http://efloraindia.nic.in/efloraindia). The plant was identified by Professor Subrata Mondal, Plant Biosystematics Laboratory, Department of Botany, Visva-Bharati and deposited to the Herbarium of Department of Botany, Visva-Bharati. Fresh leaf samples were subjected for isolation of endophytic fungi.

### Isolation and characterization of endophytic fungi

At first fresh leaves were washed thoroughly in running tap water and then surface sterilized with 4% sodium hypochlorite for 2–3 min and washed with sterilized distilled water for at least three times. Leaf samples were finally washed with 70% alcohol. After evaporation of the alcohol from leaf surfaces, they were cut into small pieces (0.5 cm × 0.5 cm) and 300 leaf segments were placed on malt extract (ME) agar plates. Four leaf segments were placed on each plate. 150 μg/ml of streptomycin was incorporated with agar plates for avoiding any possible bacterial contamination. Effectiveness of surface sterilization process was checked according to the method described by Schulz et al. [14]. After 3–4 days of incubation at 28°C, the fungal mycelia emerged from leaf samples were taken, purified by streaking on ME agar plates and finally were kept in ME agar slants at 4°C for further studies. All the fungal isolates were characterized morphologically by light microscope (Olympus CH2Oi) after staining with cotton blue and lacto phenol.

### Screening of the endophytic isolates for antibacterial properties

For selecting the potent endophytic fungus with antibacterial activities, all the endophytic isolates were grown in 100 ml of ME broth in 250 ml Erlenmeyer flask for four weeks at 28°C with mild shaking (120 rpm) in dark condition. At an interval of seven days, two ml of each fungal culture was withdrawn. Cell free supernatant (CFS) was prepared by filtration using fine muslin cloth followed by centrifugation at 10000 rpm for 10 min. Antibacterial activities of the CFS were checked using agar well diffusion method [15] both against Gram positive (*Bacillus subtilis* MTCC 121, *Listeria monocytogenes* MTCC 657, *Staphylococcus aureus* MTCC 96 and *Staphylococcus epidermidis* MTCC 2639), and Gram negative (*Salmonella typhimurium* MTCC 98, *Pseudomonas aeruginosa* MTCC 741 and *Escherichia coli* MTCC 1667) bacteria. 50 μl of culture filtrate was added to the wells (5 mm in diameter) of nutrient agar plates inoculated with test microorganisms and incubated at their respective growth temperatures (28°C or 37°C). After 24 h, zones of inhibition were observed and diameters were measured.

### Molecular identification of the potent isolate AE1

The potent endophytic fungal strain AE1 having very good antibacterial activity, was further identified on the basis of D1/D2 region of LSU (Large subunit 28S rDNA) gene sequence. D1/D2 region of 28S rDNA was amplified using forward (NL4-GGTCCGTGTTTCAAGACGG) and reverse (NL1- GCATATCAATAAGCGGAGGAAAAG) primers. Bi-directional DNA sequencing reaction was carried out with same primers using BDT v3.1 Cycle sequencing kit on ABI 3730Xl Genetic Analyzer. Alignment editor program BioEdit (http://www.nbio.ncsu.edu/BioEdit/bioedit.html) was used to generate nucleotide sequence of D1/D2 region of 28S rDNA from forward and reverse sequence data. Nucleotide sequence was used to carry out BLAST with the nr database of NCBI GenBank database (http://www.ncbi.nlm.nih.gov/). Based on maximum identity score, first fifteen sequences were selected and evolutionary relationship was established using the Neighbor-Joining method [16]. Phylogenetic tree was constructed using MEGA5 [17]. The evolutionary distances were computed using the Kimura 2-parameter model [18]. *Cladosporium cladosporioides* CBS 170.54 (AY213694.1) was used as an out group member during phylogenetic analysis.

### Determination of nature of antibacterial principle

To find out the thermostability of antibacterial principle the CFS of AE1 was kept in boiling water bath for 10 min. In addition, to check its protinaceous nature, the CFS was treated with protinase K at a concentration of 1 mg/ml for 2 h at 37°C. Antibacterial activity of both heat treated and protinase K treated CFS were determined by agar well diffusion method [15] against the bacterial organisms in the presence of appropriate control.

### Extraction of endophytic fungal (AE1) metabolites

On the basis of maximum antimicrobial metabolites production in ME broth, it was selected for the solvent extraction process. The CFS of the potent endophytic fungal isolate AE1 was subjected to ethyl acetate (EA) extraction. AE1 was inoculated in 1L of ME broth in 2L Erlenmeyer flasks and incubated at 28°C in dark condition. After 21 days, broth containing fungal growth was taken and filtered with muslin cloth. CFS was prepared by centrifugation at 10000 rpm for 10 min and extracted thrice with EA. For every 100 ml of CFS, 40 ml of EA was added, mixed vigorously and the EA layer was separated using separating funnel. The EA portion was then evaporated to dryness using rotary vacuum evaporator at room temperature (26±2°C), weighed and stored at 4°C for further experiments.

### Study of antibacterial activities of EA fraction of AE1

Antibacterial activities of EA fraction of the CFS of AE1 were checked against both Gram positive and Gram-negative organisms using disc diffusion method [19]. Different concentrations (500 μg/ml-5 mg/ml) of EA fraction were prepared after dissolving it in dimethyl sulphoxide (DMSO). 10 μl of each concentration was added to sterilized filter paper discs and air dried. The discs containing different concentrations of EA fraction (5–50 μg/disc) were then placed on the lawn of tested bacteria on nutrient agar plates. Plates were incubated at their respective growth temperatures (28°C or 37°C). Zones of inhibition were observed after 24 h and diameters were measured. The broad spectrum antibiotic Streptomycin at a concentration of 1 μg/disc was used as positive control during this study.

Antibacterial activity of EA fraction of AE1 was also studied by counting the numbers of colony forming units (CFUs) of these tested bacteria after treatment. In fresh nutrient broths 1% of each bacterial culture (OD_620nm_ = 0.5) was inoculated and treated with different concentrations (100–500 μg/ml) of EA extract of AE1. Untreated bacterial culture was considered as control set. Treated and untreated sets were incubated for 24 h at their respective growth temperatures. CFUs were counted by spreading on nutrient agar plates after proper dilution and compared with their untreated control.

The mode of action of the antimicrobial metabolites present in the EA fraction of AE1 on pathogenic bacteria was studied by adding it to the actively growing cultures of one Gram positive bacterium *Listeria monocytogenes* MTCC 657 and one Gram negative bacterium *Escherichia coli* MTCC 1667 at concentrations of 300 μg/ml and 400 μg/ml respectively. At an interval of two hours, the numbers of CFUs were counted and presented graphically to observe the growth inhibition patterns.

### Scanning electron microscopic studies

The effect of EA extract of AE1 on the bacterial cells was checked by scanning electron microscopic (SEM) studies. Both the treated and untreated bacterial cells were harvested by centrifugation at 6000 rpm for 10 min. Pellets were smeared on clean glass slide (1 cm × 1 cm), air dried and heat fixed. Samples were pre-fixed with 2% glutaraldehyde in 20 mM sodium- phosphate (Na-P) buffer (pH 6.5) plus 5% DMSO for 30 min. Then the samples were washed with 20 mM Na-P buffer (pH 6.5) and postfixed with osmium tetraoxide dissolved in 50 mM Na-P buffer (pH 6.5). Bacterial cells were then dehydrated using a series of alcohol grades (30%-100%), retaining them for 10 min in each grade. The dehydrated cells were coated with gold using an ion sputter (Coater IB-2, Gikeengeering, Japan) and finally observed under scanning electron microscope (Zeiss) following the standard method [20].

### Study of the release of intracellular materials of bacterial cells

In order to ascertain the effects of EA fraction on bacterial cell integrity or membrane permeability, extracellular concentrations of nucleic acids, protein, and potassium ion (K^+^) were determined after treatment with EA fraction. Gram positive bacterium *L*. *monocytogenes* and Gram negative bacterium *E*. *coli* were grown (OD_620nm_ = 0.5) in 200 ml of NB in 500 ml Erlenmeyer flask and cells were harvested by centrifugation (6000 rpm). Cells were washed using 50 mM Na-P buffer and re-suspended in one ml of the same buffer. Then they were treated with EA extract of AE1 at their respective MIC values for 6 h and 24 h. The supernatants were taken by centrifugation (10000 rpm) and used for quantification purpose. Untreated set for each bacterium was used as control. Concentration of DNA and proteins were determined following the methods of Burton [21] and Lowry et al. [22] respectively. On the other hand, K^+^ ion concentrations were determined with the help of flame photometer (Elico, CL-378) using K_2_HPO_4_ as standard.

### Study of the effect of EA fraction on bacterial key enzymes

Cells of four different bacteria were treated with sub lethal (250 μg/ml and 350 μg/ml for Gram positive and Gram negative bacteria respectively) and lethal (300 μg/ml and 400 μg/ml for Gram positive and Gram negative bacteria respectively) concentrations of the EA extract of AE1 for 24 h. Cells were harvested by centrifugation at 6000 rpm for 10 min, washed with sterilized Na-P buffer solution (20 mM) and were suspended in one ml of the same buffer. Cells were ruptured for getting cell free extract (CFE) following the method described by Debnath et al. [23] using sonicator (LABSONIC M). Supernatants were collected by centrifugation at 10000 rpm for 10 min and used as crude enzyme. Three enzymes viz., phosphorfructokinase (PFK), isocitrate dehydrogenase (ICDH) and fructose-1,6-bisphosphatase (FBPase) were assayed in this study following the method described by Mandal et al. [24]. Substrate, co factor and other requirements mixed in a cuvette, respective CFEs as crude enzymes were added and rate of reduction of NADP was followed at 340 nm in a UV-Vis spectrophotometer (SHIMADZU UV1700). Specific activity was calculated as nano moles of substrate consumed per min per mg protein and compared to untreated control.

### Thin layer chromatographic analysis of EA fraction

EA fraction of AE1 was subjected to thin layer chromatographic (TLC) analysis for partial separation of active metabolites. Dried extract was re-dissolved in EA at a concentration of 20 mg/ml and 10 μl of solution was spotted on TLC plates (MERCK Silica gel F254). EA and chloroform in a ratio of 1:1 was used as running solvent. Under UV light, retention factor (R_f_) values for all the bands were calculated. The compounds related to each band were collected by scratching and dissolving in one ml of EA. EA portion was separated by centrifugation at 6000 rpm for 10 min and was evaporated to dryness. After dissolving the dried compounds in DMSO at 100 μg/ml concentration, antibacterial activities were determined by disc diffusion method [19]. Zones of inhibition were observed and diameters were measured.

### GC-MS analysis of antibacterial principles

In order to characterize the antibacterial compounds, the active fractions derived after TLC analysis were subjected for Gas Chromatograohy-Mass Spectromatry (GC-MS) analysis. This was carried out using TR-Wax GC Column (Thermo Fisher Scientific) following ion trap technology. Helium gas at a flow rate of 1 ml/min was used as carrier and an injection volume of 2 μl was used (spit ratio 10:1). Injection port base temperature was maintained at 250°C. The oven was initially heated at 50°C for 2 min and increased upto 280°C and maintained for 9 min. The ionizing voltage of 70 eV was used for Mass spectra. The identification of bioactive compounds were performed by comparing the mass spectra with data from NIST05 (National Institute of Standard and Technology, US) library.

### Evaluation of antioxidant activities

#### DPPH free radical scavenging assay

Different concentrations of EA fraction of AE1 were tested for antioxidant activities using 2, 2-Diphenyl-1-picrylhydrazyl (DPPH) free radical scavenging assay [25] and compared with well known antioxidant ascorbic acid. The dried fraction was dissolved at a concentration of 10 mg/ml in methanol. 100 μl of various concentrations of methanolic fractions were mixed with 2900 μl of DPPH solution and incubated for 30 min at room temperature. In control set, only 100 μl of methanol was added to the DPPH. The absorption was recorded at 517 nm and the percentage of inhibition (%I) of free radicals was calculated as: %I = [(A blank-A sample)/A blank] ×100, where, A blank: Absorbance of control, A sample: Absorbance of methanolic solution and DPPH. By plotting straight line equation, IC_50_ value was calculated.

#### Superoxide radical scavenging assay

Superoxide radical scavenging activity of the EA fraction was determined by using phenazine methosulfate-nicotinamide adenine dinucleotide (PMS/NADH) system [26]. This system generates superoxide radicals that reduce nitro blue tetrazolium (NBT) and produces purple color formazan. One ml of reaction mixture was prepared by adding Na-P buffer (20 mM, pH 7.4), NADH (73 *μ*M), NBT (50 *μ*M), PMS (15 *μ*M) and EA fraction of AE1 (0–250 μg/ml). After five min of incubation at room temperature (25°C), OD was taken at 562 nm using UV-Vis spectrophotometer (SHIMADZU UV-1700, Japan) in the presence of appropriate blank. Quercetin was used as positive control. The inhibition percentage and the IC_50_ value were calculated as described above for DPPH free radical scavenging assay.

### Statistical analysis

All the experiments were repeated and the data represented are means of at least three replicates. For calculating means and standard deviation, Microsoft Excel program version 2007 was used.

## Results and discussions

### Selection and characterization of potent endophytic fungal strain

**Fig 1:**
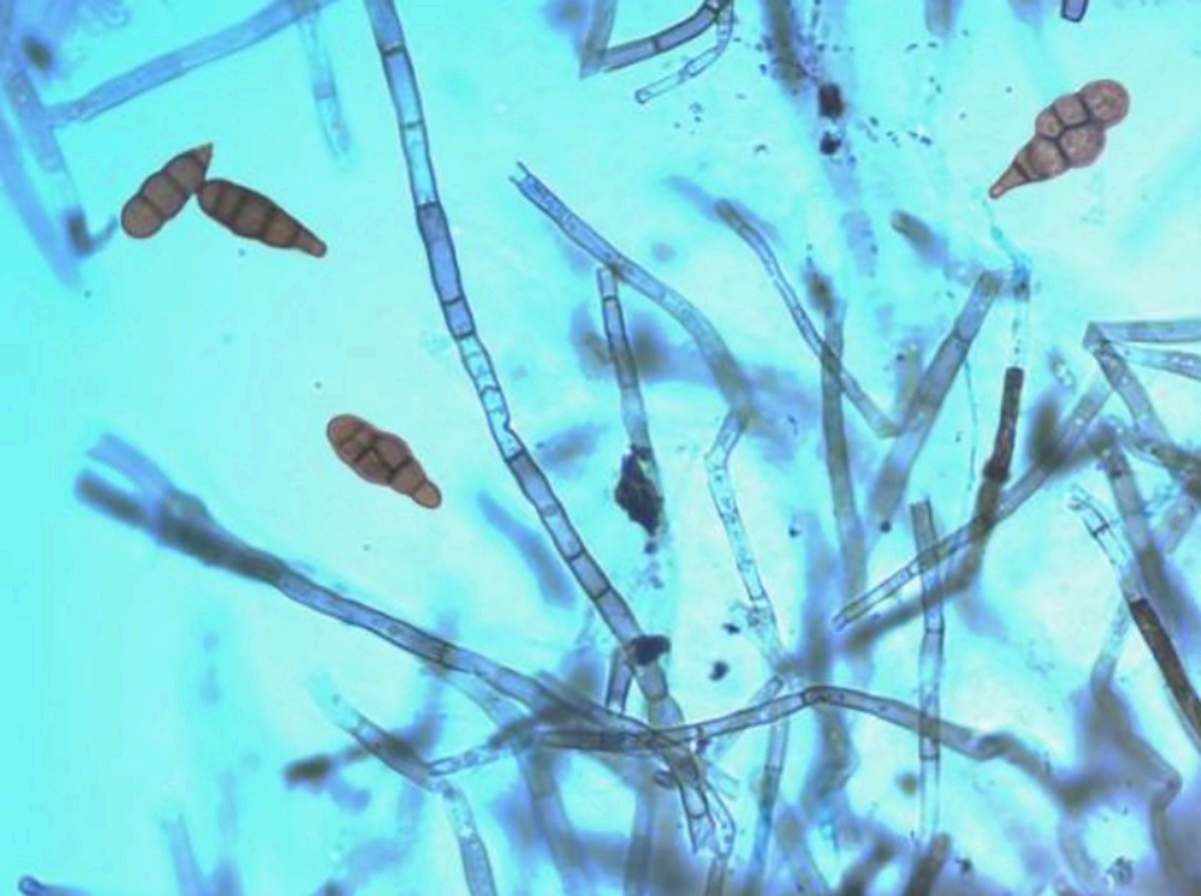
Morphology of endophytic fungus AE1 under light microscope.

In the present endeavor, the well known medicinal plant *Azadirachta india* A. Juss. was subjected for the isolation of endophytic fungi. From healthy and mature leaves of *A*. *indica*, nine different fungal strains with different colony morphologies were isolated. Based on morphological characteristics under light microscope, five of them were identified as species of *Alternaria* and two of them were identified as species of *Colletotrichum*. Other two fungal strains remain unidentified due to lack of any reproductive structures. When the antibacterial potential of these nine fungal strains were tested against a number of pathogenic bacterial strains, the strain *Alternaria* sp. AE1 was found more effective than the other strains in terms of antimicrobial activities. The isolate AE1 showed white cottony spreading colony which became brownish gray upon maturation. It produced multicellular beaked conidia having both transverse and longitudinal septations (Fig 1). The CFS of AE1 prepared from inoculated broth after seven days of incubation, was able to produce prominent zones of inhibition against all the Gram-positive bacteria as well as Gram-negative bacteria except *P*. *aeruginosa*. Verma et al. [10] also reported *Alternaria* sp. having antimicrobial potential from *A*. *indica*. On the basis of the antibacterial potential among the nine strains, the strain AE1 was considered for further studies.

### Molecular identification of AE1

**Fig 2:**
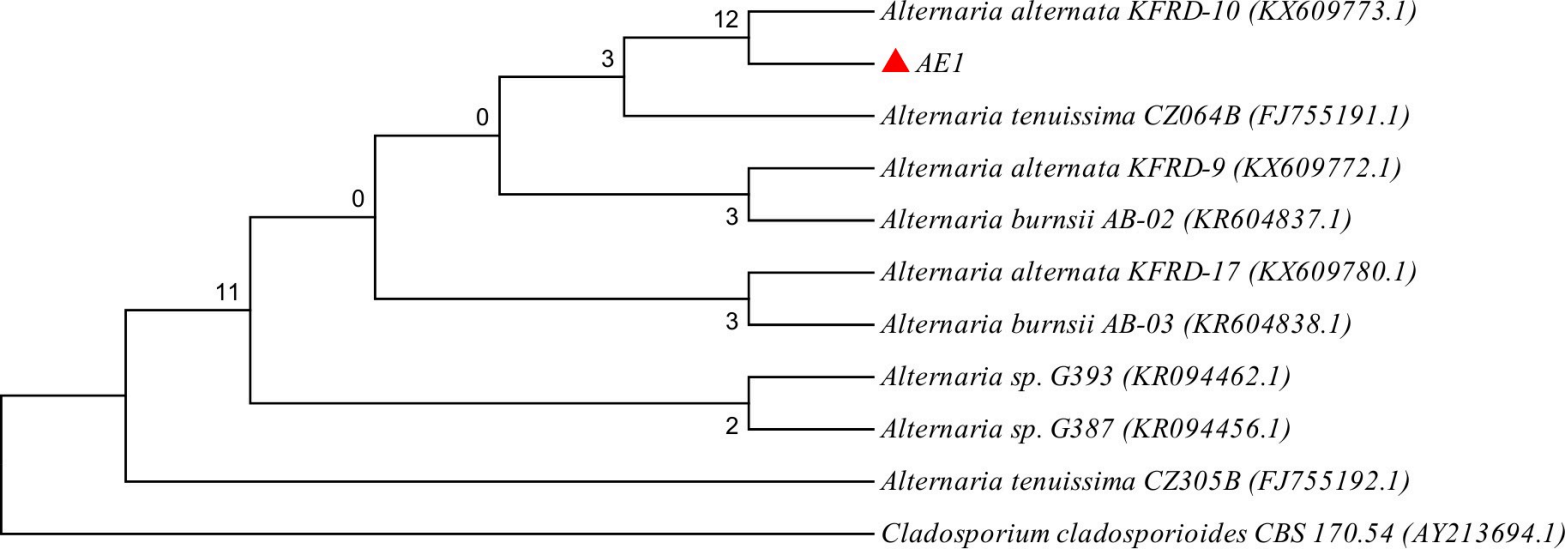
Neighbor joining phylogenetic tree on the basis of 28S rDNA (D1/D2 region) sequence of AE1.

PCR amplification and sequencing of D1/D2 region of 28S rDNA of AE1 revealed a 645 bp nucleotide sequence which showed 99% pair wise similarity with *Alternaria alternata* KFRD-10. The neighbor joining phylogenetic tree also showed its close relationship with that strain (Fig 2). On the basis of BLAST as well as phylogenetic analysis the strain AE1 was identified as *Alternaria alternata*. *Alternaria alternata* isolated from *Camellia sinensis*, has been reported as an potent endophytic fungus producing numbers of bioactive compounds [27].

### Nature of antibacterial principle

Like untreated CFS, prominent zones of inhibitions against all tested bacterial organisms were observed for both heat killed as well as proteinase K treated CFS of AE1. No zones of inhibition were observed in control (uninoculated broth and 1 mg/ml of proteinase K). Zones of inhibition produced by both heat killed and Proteinase K treated CFS indicated thermostable and non-proteinacious nature of the antibacterial principle(s). Wang et al. [27] has reported production of two altenuene derivatives, one isocoumarin along with six other compounds by *Alternaria alternata*.

### Antibacterial activities of EA fraction of AE1

By considering the non-proteinacious nature of the antimicrobial principle(s), the CFS of AE1 was subjected for solvent extraction. Among the three different culture media viz. ME broth (MEB), potato dextrose broth (PDB) and Czapek dox broth (CDB), maximum production of antimicrobial principles was found in the presence of ME broth (S1 Fig) and therefore it was subjected for solvent extraction. Although different solvents were used initially for the extraction purpose, the EA was selected as the suitable one for its maximum efficacy to extract the bioactive compounds produced by the isolate. When the antibacterial potential of the EA fraction of AE1 was determined at different concentrations following disc diffusion method [19], prominent zones of inhibition were observed against all the Gram positive and two Gram negative bacteria (Table 1) tested. Maximum diameter of zones of inhibition was found in case of Gram positive bacteria than Gram negative bacteria (Table 1). Like CFS, the EA fraction was also unable to produce zone of inhibition against the Gram negative bacterium *P*. *aeruginosa*. The probable reason for maximum activity of the extract against Gram positive bacteria may be due to the basic difference of the cell wall properties of these bacteria. In Gram negative bacteria, the outer layer of the cell wall is composed of lipopolysachharide which makes the cell wall impermeable for lipophilic solutes. On the other hand the peptidoglycan present in Gram positive cell wall is not a permeability barrier of lipophilic solutes [28]. Hellwig et al. [29] also isolated a natural product Altersin from two endophytic *Alternaria* species which also showed similar antimicrobial potential against pathogenic Gram-positive bacteria.

**Table 1:** Antibacterial activity of ethyl acetate extract of AE1 by disc diffusion method.

Concentrations of EA fraction (μg/disc)	Diameter of inhibition zones (mm) against pathogenic bacteria				
	*B*. *subtilis*	*L*. *monocytogenes*	*S*. *aureus*	*E*. *coli*	*S*. *typhimurium*
DMSO(Control)	0	0	0	0	0
5	8 ± 1.5	9 ± 1.5	8 ± 1.0	7 ± 1.0	8 ± 1.0
10	10 ± 1.5	10 ± 1.0	10 ± 1.0	8 ± 1.0	9 ± 1.5
20	11 ± 1.0	11 ± 1.5	10 ± 1.5	8 ± 1.5	10 ± 1.5
30	12 ± 2.0	12 ± 1.5	10 ± 1.0	9 ± 1.0	10 ± 2.0
40	13 ± 1.0	13 ± 1.0	11 ± 1.5	9 ± 1.5	10 ± 2.0
50	14 ± 1.5	14 ± 1.5	12 ± 1.0	11 ± 1.0	12 ± 1.5
Streptomycin (1 μg/disc)	20 ± 1.0	18 ± 1.5	14 ± 1.0	16 ± 1.0	18 ± 1.5

### Effect of EA fraction of AE1 on bacterial CFUs

When the effect of EA fraction was tested by counting the bacterial CFUs, the number of CFUs markedly reduced upon treatment with increased concentrations of EA fraction of AE1 especially in case of Gram positive bacteria. As the EA fraction showed sufficient reduction (almost zero) in the bacterial CFUs at concentrations of 300 μg/ml and 400 μg/ml against Gram positive and Gram negative bacteria respectively, therefore these concentrations can be considered as MIC values of the EA fraction (Table 2). The EA extract of endophytic fungus *Alternaria alternata* VN3 isolated from *Vitex negundo* L. was also effective against both Gram positive and Gram negative bacteria and it showed MIC values ranging from 100 to 900 μg/ml for *Staphylococcus aureus* and *Pseudomonas aeruginosa* [30]

**Table 2:** Effect of ethyl acetate fraction of AE1 on CFUs of pathogenic bacteria.

Conc. of EA fraction (μg /ml)	CFU/ml of pathogenic bacteria				
	*B*. *subtilis*	*L*. *monocytogenes*	*S*. *aureus*	*E*. *coli*	*S*. *typhimurium*
Control	7.52 × 10^7^	3 × 10^8^	8.43 × 10^9^	4.92 × 10^8^	5.06 × 10^8^
100	6.2 × 10^5^	3.8 × 10^6^	6.87 × 10^7^	2.18 × 10^7^	3.2 × 10^7^
200	2.56 × 10^2^	1.8 × 10^2^	2.47 × 10^3^	2.2 × 10^5^	6.0 × 10^4^
300	0.2 × 10^1^	0	0	6.6 × 10^2^	8.4 × 10^2^
400	0	0	0	0.32 × 10^1^	0
500	0	0	0	0	0

### Mode of action of EA fraction of AE1

**Fig 3:**
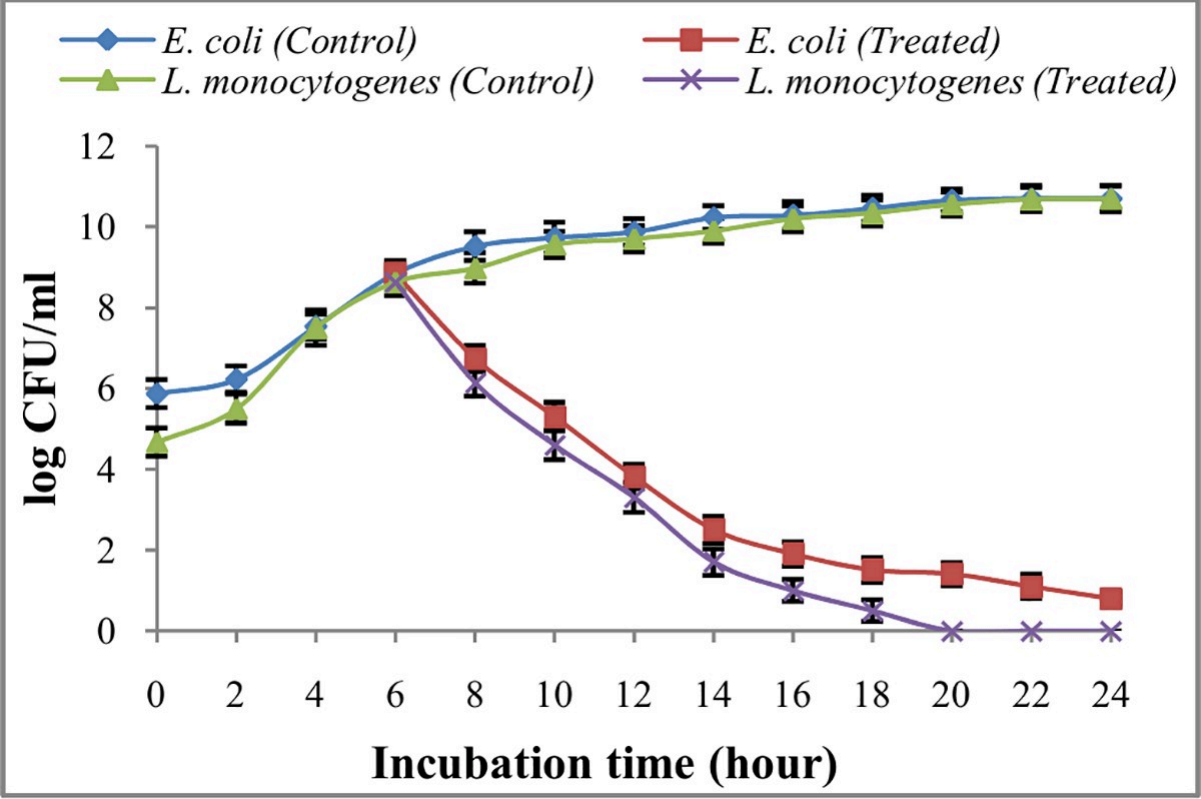
Mode of action of ethyl acetate fraction of AE1 on bacterial growth.

In order to find out the mode of action of extracted compounds against pathogenic bacteria, EA fraction of AE1 was added at a concentration of 300 μg/ml and 400 μg/ml to the mid log phase cultures of *L*. *monocytogenes* and *E*. *coli* respectively. Drastic reductions in the number of CFUs with time upon treatment were observed for both the bacteria tested (Fig 3). In treated sets, the CFUs especially in case of *L*. *monocytogenes* became almost zero after 20 h of incubation. This type of drastic reduction in the number of CFUs deep rooted strong cidal mode of action of the antibacterial principle(s). The reduction in the number of CFUs also indicated the better activities of the antimicrobial principle(s) against Gram positive organism than Gram negative one (Fig 3).

### SEM studies of pathogenic bacterial cells

**Fig 4:**
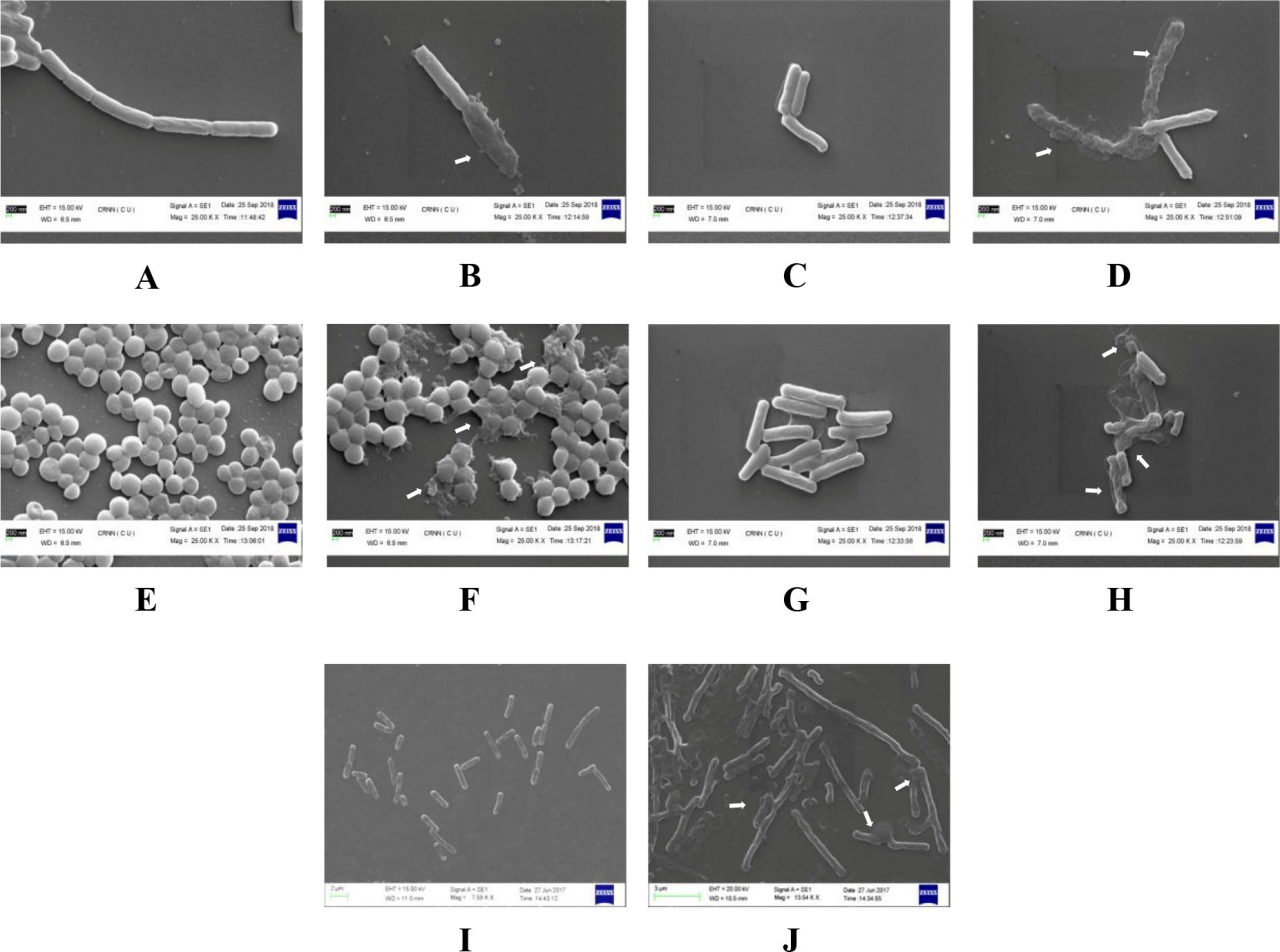
Effect of ethyl acetate fraction of AE1 on the morphology of pathogenic bacteria found during SEM studies: A- *B*. *subtilis* (untreated); B- *B*. *subtilis* (treated); C- *L*. *monocytogenes* (untreated); D- *L*. *monocytogenes* (treated); E- *S*. *aureus (*untreated); F- *S*.*aureus* (treated); G- *S*. *typhimurium* (untreated); H- *S*. *typhimurium* (treated); I- *E*. *coli* (untreated); J- *E*. *coli* (treated).

During SEM study, prominent degradation of bacterial cell wall structures was noticed for all the pathogenic bacteria tested. Among the Gram positive bacteria, maximum deformations were observed in *B*. *subtilis* (Fig 4B) and in *L*. *monocytogenes* (Fig 4D) in comparison to *S*. *aureus* (Fig 4F). Deformations of cell structures were also observed in Gram negative *S*. *typhimurium* (Fig 4H) and in *E*. *coli* (Fig 4J). In addition to cell wall degradation, end to end joining of cells was observed in treated *E*. *coli* (Fig 4J). Massive distortion of bacterial cell morphologies as observed by SEM study suggested the probable effect of antimicrobial compounds on bacterial cell wall. End to end joining or mycelia nature of *E*. *coli* upon treatment indicated an effect of antimicrobial compounds on normal cell division function [31].

### Release of intracellular materials of bacterial cells

**Fig 5:**
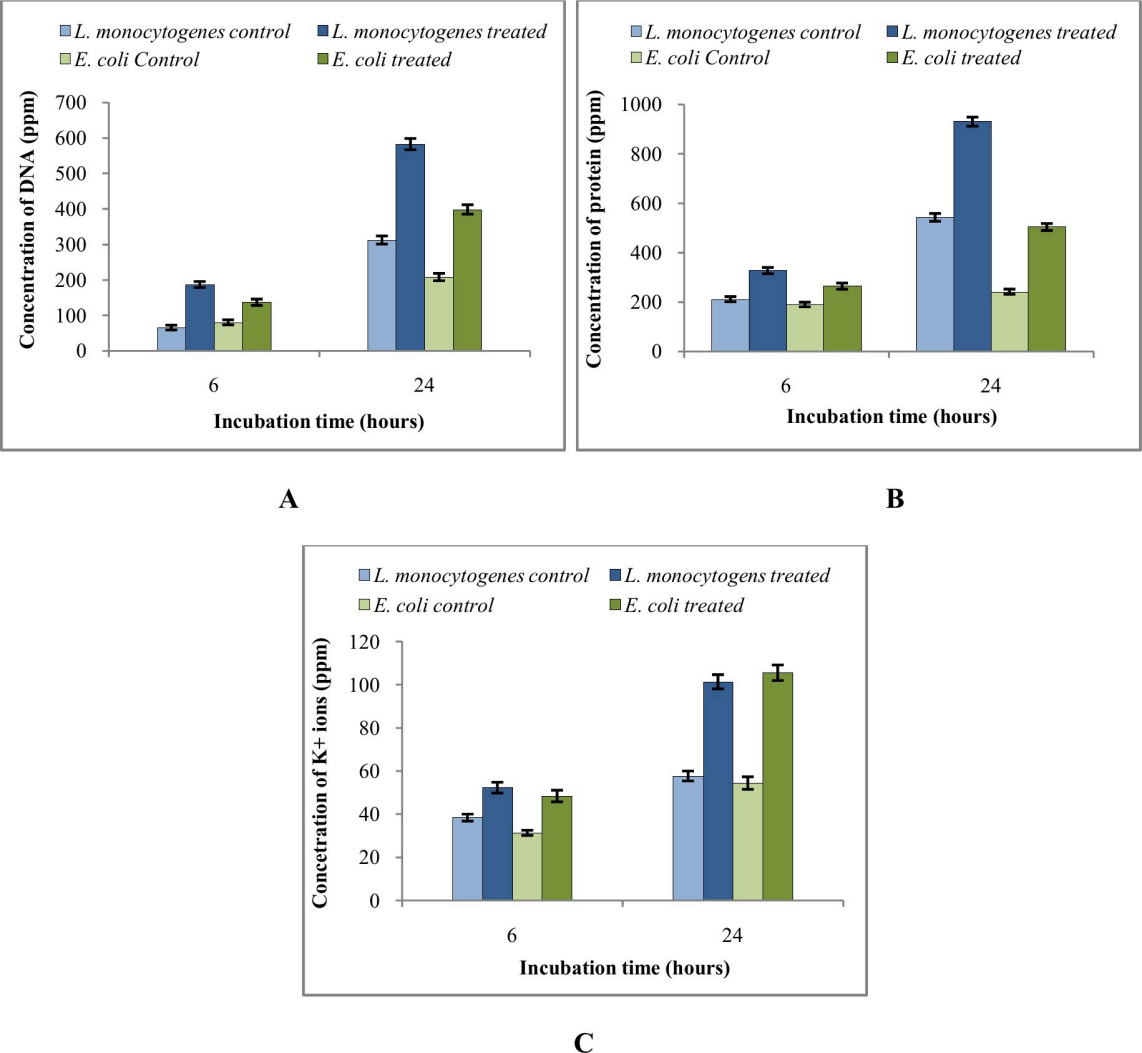
Release of intracellular materials from pathogenic bacterial cells upon treatment with EA fraction of AE1: A- release of DNA; B- release of protein; C-leakage of K^+^ ion.

Upon treatment with EA fraction of AE1, release of DNA, protein and K^+^ ion was observed both in Gram positive *L*. *monocytogenes* and in Gram negative *E*. *coli*. Quantification of DNA, protein and K^+^ ion in the extracellular environment suggested their release from bacterial cells within 6 h of treatment. Almost two fold increase in DNA and protein concentrations was found after 24 h of incubation in treated set especially for Gram positive *L*. *monocytogenes* (Fig 5A & 5B). It was also observed that the antimicrobial metabolites were more effective on Gram positive *L*. *monocytogenes* than Gram negative *E*. *coli*. Release of DNA and protein from bacterial cells suggested lysis of bacterial cells upon treatment. Increase of K^+^ ion (Fig 5C) also indicated membrane leakage in treated cells [32]. These observations also confirmed the degradation of bacterial cell structures upon treatment as observed by SEM study.

### Effect of EA fraction of AE1 on central carbohydrate metabolism of bacteria

**Fig 6:**
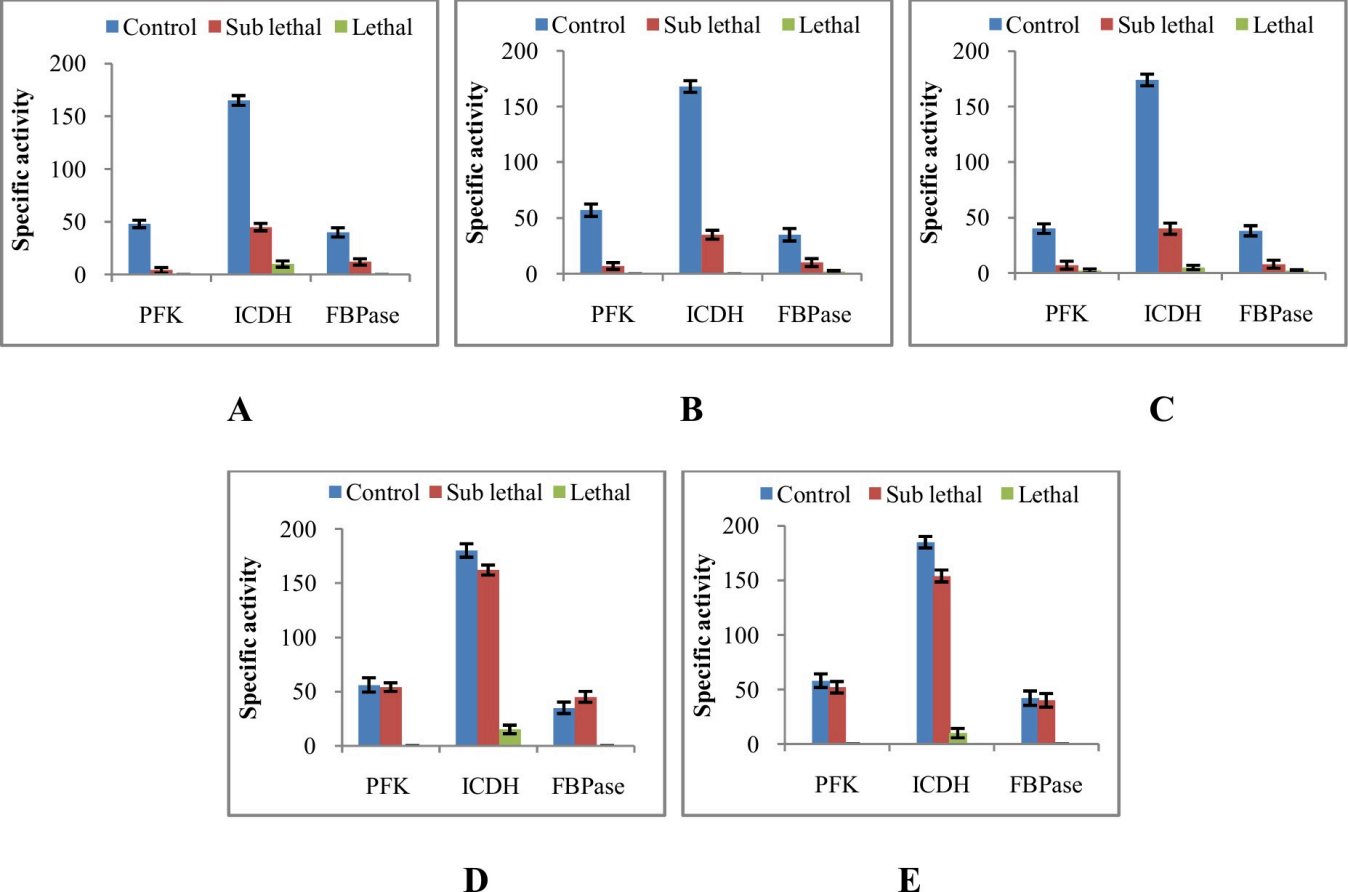
Profile of central carbohydrate metabolic enzymes in pathogenic bacteria upon treatment with ethyl acetate extract of AE1: A- *B*. *subtilis*; B- *L*. *monocytogenes*; C-*S*. *aureus*; D- *E*. *coli*; E- *S*. *typhimurium*.

At lethal concentrations the extract of AE1 could reduce drastically the activity of central carbohydrate metabolic pathway enzymes viz. PFK, ICDH and FBPase in all the bacteria tested. However there was almost no change of these metabolic enzymes in Gram negative bacteria especially in *E*. *coli* and *S*. *typhimurium* at sub lethal concentration whereas in Gram positive bacteria these activities at sub lethal concentration were reduced greatly (Fig 6A–6E). This observation indicated its direct or indirect interference with energy metabolism via central carbohydrate metabolic pathways [33]. Slight increase in FBPase activity was noticed in Gram negative bacteria treated with sublethal concentration. This indicated a struggle and overcome of the stress by the treatment of the compound which triggered the induction of gluconeogenic FBPase. Activation of some key enzymes in stressed condition has also been reported in *Rhizobium* and in Cyanobacteria by previous workers [34,23]. As at lethal concentrations the organisms were unable to overcome the stress conditions, therefore the FBPase activity decreased significantly (6D-E).

### Thin layer chromatographic analysis

**Fig 7:**
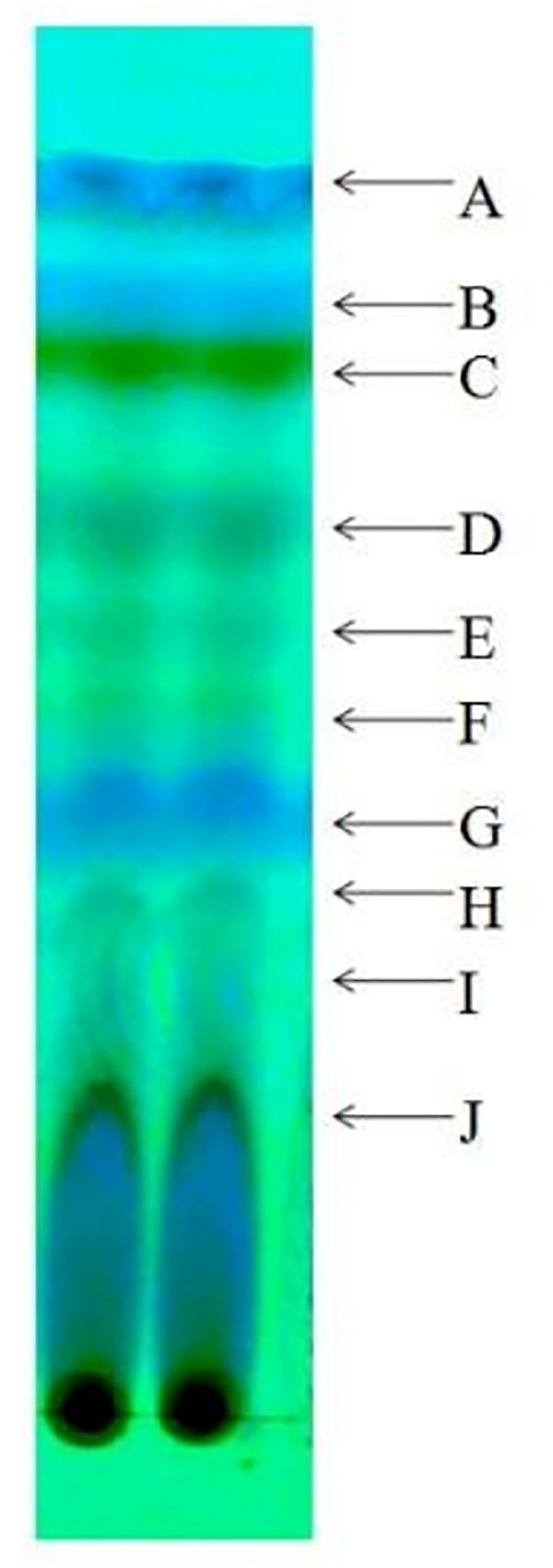
Thin layer chromatographic analysis of ethyl acetate fraction of AE1.

**Fig 8:**
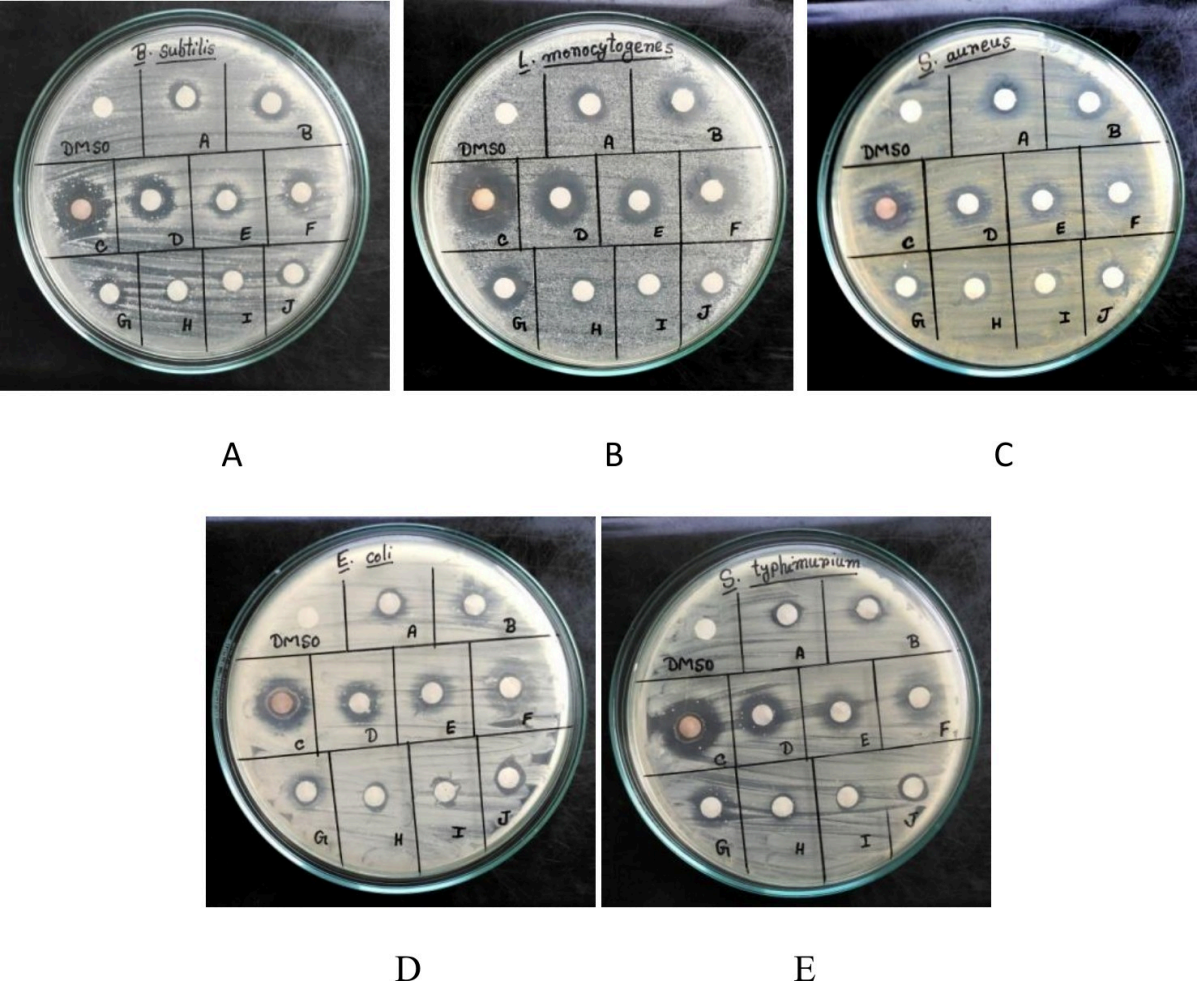
Zones of inhibition produced by compounds derived from TLC plate, against pathogenic bacteria A- *B*. *subtilis;* B- *L*. *monocytogenes*; C- *S*. *aureus*; D- *E*. *coli*; E- *S*. *typhimurium*.

TLC analysis of EA extract of AE1 revealed ten separate bands under UV light (Fig 7). When the antibacterial activities of the bands were checked, seven bands out of ten were effective to produce zones of inhibition against tested bacterial organisms (Fig 8). Among them, band C (Rf value 0.77) and band D (Rf value 0.61) showed maximum zones of inhibition with large zone diameter against three Gram positive and two Gram negative bacteria tested (Fig 8). Appearance of seven different bands on TLC plate having antimicrobial activities indicated the production of different bioactive compounds by the single isolate AE1. Production of numbers of bioactive metabolites by *Alternaria alternata* has been reported earlier [27]. On the other hand, Alternariol 9-methyl ether having antimicrobial activity has also been characterized from *Alternaria* sp. Samif01 derived from *Salvia miltiorrhiza* Bunge [35].

### GC-MS analysis for the detection of bioactive compounds in EA fraction of AE1

**Fig 9:**
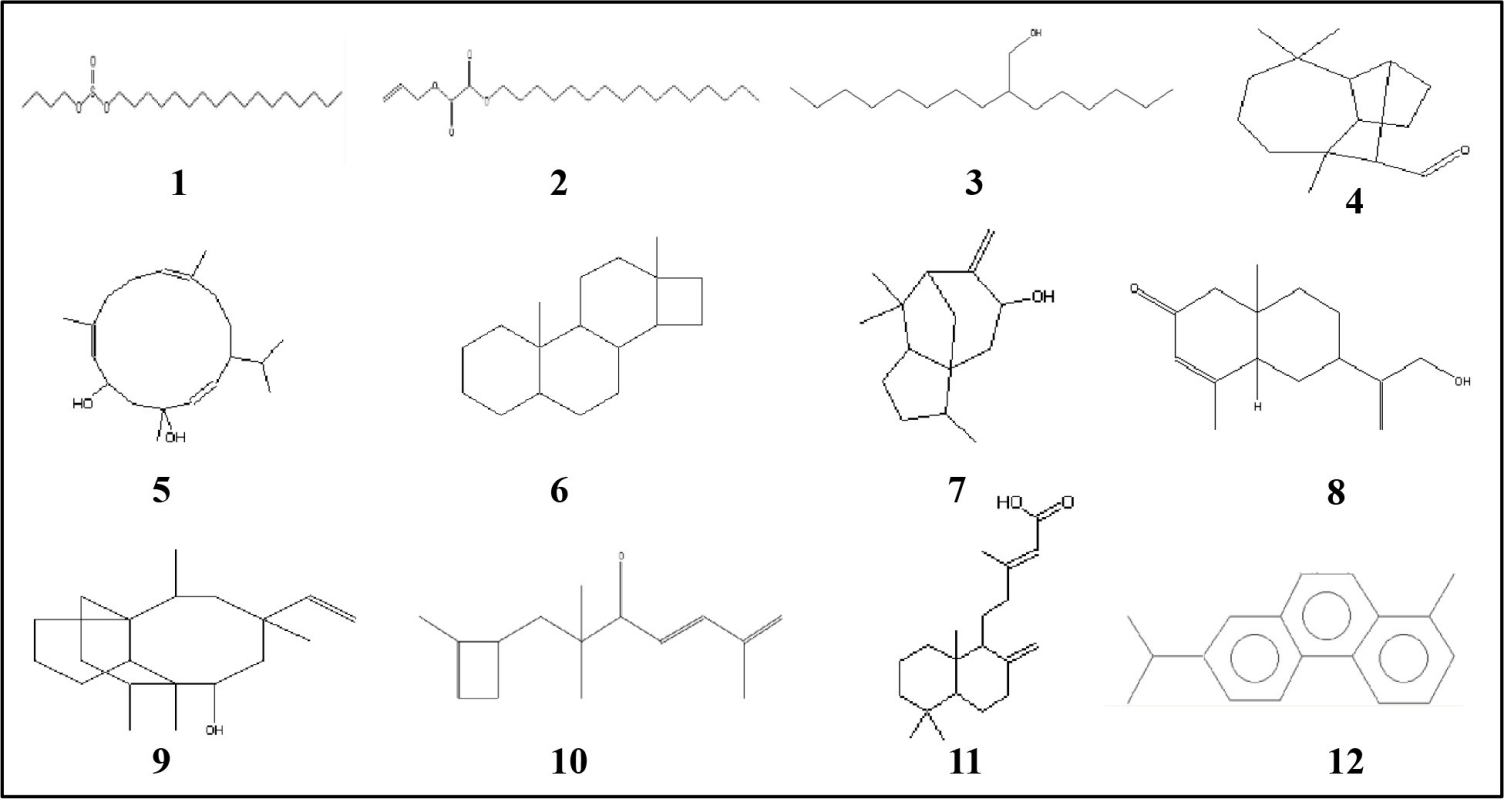
Chemical structures of twelve different compounds identified from band-C of EA fraction of AE1 by GC-MS analysis as mentioned in Table 3.

**Fig 10:**
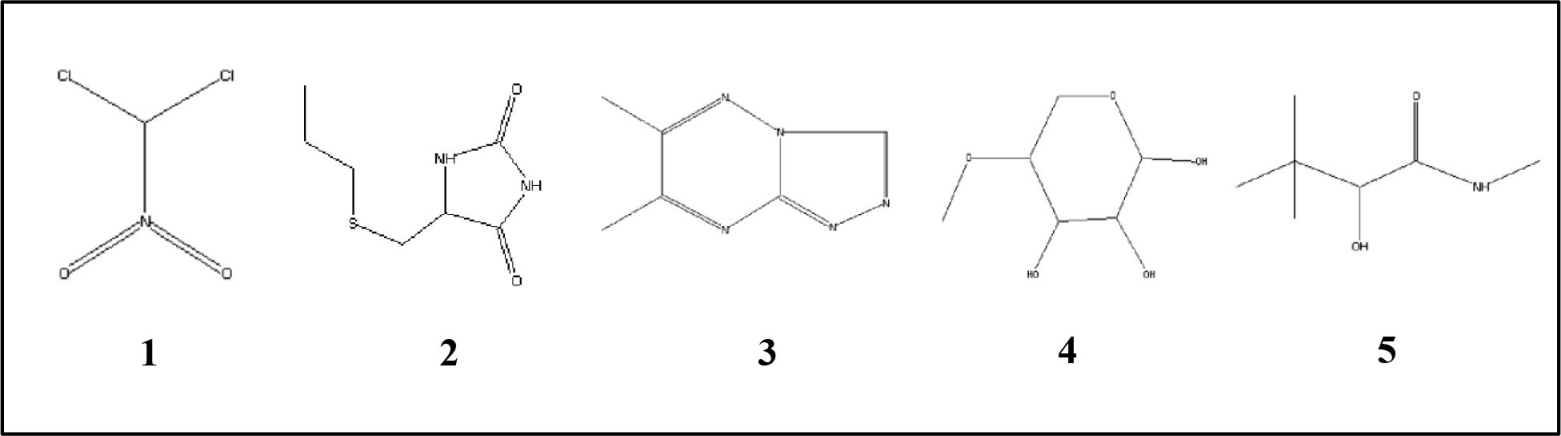
Chemical structures of five different compounds identified from band-D of EA fraction of AE1 by GC-MS analysis as mentioned in Table 4.

On the basis of TLC analysis followed by antimicrobial study, band C and band D were found as most effective in terms of antimicrobial potential and further considered for GC-MS analysis. This analysis and NIST library search revealed the presence of twelve different compounds in band C and five different compounds in band D with similarity indices of ≥90%. The retention time (RT), chemical formula, and MW are represented in Tables 3 and 4. Their chemical structures are also represented in Figs 9 and 10. Among the 12 different compounds present in band C, based on peak area percentages d-Norandrostane (5à,14à) (steroid), Longifolenaldehyde (terpene), 4,8,13-cyclotetradecatriene-1,3-diol,1,5,9-trimethyl-12-(1-methylethyl) (macrocyclic diterpene), 2,4,7,14-Tetramethyl-4-vinyl-tricyclo[5.4.3.0(1,8)]tetradecan-6-ol, 1H-3a,7-Methanoazulen-5-ol, octahydro-3,8,8-trimethyl-6-methylene (terpene) were found as dominant members. On the other hand in case of band D, Dichloronitromethane was detected as dominant compound. Govindappa et al. [36] also reported numbers of bioactive compounds from endophytic *Alternaria alternata* isolated from *Tabebuia argentea*.

**Table 3:** Compounds identified from band-C of EA fraction of AE1 by GC-MS analysis.

No.	RT	Name of the compound	Molecular formula	MW	Peak Area %
1.	18.90	Sulfurous acid, butyl heptadecyl ester	C_21_H_44_O_3_S	376	0.51
2.	19.73	Oxalic acid, allyl hexadecyl ester	C_21_H_38_O_4_	354	3.65
3.	20.81	2-Hexyl-1-decanol	C_16_H_34_O	242	4.75
4.	26.06	Longifolenaldehyde	C_15_H_24_O	220	8.93
5.	26.22	4,8,13-cyclotetradecatriene-1,3-diol,1,5,9-trimethyl-12-(1-methylethyl)	C_20_H_34_O_2_	306	7.86
6.	28.58	d-Norandrostane (5à,14à)	C_18_H_30_	246	10.02
7.	28.88	1H-3a,7-Methanoazulen-5-ol, octahydro-3,8,8-trimethyl-6-methylene-	C_15_H_24_O	220	5.32
8.	29.14	6-[1-(hydroxymethyl)vinyl]-4,8a-dimethyl-4a,5,6,7,8,8a-hexahydro-2(1H)-napthalenone	C_15_H_22_O_2_	234	2.39
9.	29.59	2,4,7,14-Tetramethyl-4-vinyl-tricyclo[5.4.3.0(1,8)]tetradecan-6-ol	C_20_H_34_O	290	7.48
10.	30.86	2,2,6-Trimethyl-1-(2-methyl-cyclobut-2-enyl)-hepta-4,6-dien-3-one	C_15_H_22_O	218	1.12
11.	30.99	Labda-8(20), 13-dien-15-oic acid	C_20_H_32_O_2_	304	4.25
12.	38.59	Phenanthrene, 7-isopropyl-1-methyl (Retene)	C_18_H_18_	234	3.34

**Table 4:** Compounds identified from band-D of EA fraction of AE1 by GC-MS analysis.

No.	RT	Name of the compound	Molecular formula	MW	Peak Area %
1.	2.05	Dichloronitromethane	CHCl_2_NO_2_	129	2.8
2.	3.38	L-5-Propylthiomethylhydantoin	C_7_H_12_N_22_S	188	0.4
3.	31.85	6,7-Dimethyl-triazolo(4,3-b)(1,2,4)-triazine	C_6_H_7_N_5_	149	0.6
4.	36.44	4-O-Methyl-d-arabinose	C_6_H_12_O_5_	164	0.42
5.	40.68	Butanamide, -hydroxy-N,3,3-trimethyl	C_7_H_15_NO_2_	145	0.3

### Antioxidant activity of EA fraction of AE1

Endophytic fungi have also been reported as a good source of antioxidant metabolites [37]. During DPPH free radical scavenging assay in the presence of different concentration of EA fraction of AE1, prominent color changes from pink to yellow of DPPH solution were observed along with increased concentration of the extract. IC_50_ value of EA fraction of AE1 was calculated as 38.0±1.7 μg/ml from the standard curve of percentages of inhibition whereas, IC_50_ value of ascorbic acid was calculated as 20.23±2.3 μg/ml which was used as positive control in this assay. The crude extract of *A*. *alternata* isolated from *Coffea arabica* L., showed an IC_50_ value of 86.7 μg/ml when tested by DPPH free radical scavenging assay [38].

Superoxide anions are very harmful and detrimental on cell functions and survival. The EA fraction of AE1 also showed a substantial superoxide free radical scavenging properties. Prominent reduction in purple colour formation was observed along with increased concentration of EA fraction. IC_50_ value of 11.38±1.2 μg/ml was calculated for EA fraction of AE1. Quercetin, which was used as a positive control showed an IC_50_ value of 42.31±1.8 μg/ml. It has been reported that different types of flavonoid compounds produced by plant or by their endophytic fungi can scavenge superoxide free radicals and act as effective antioxidant molecules [39,40].

## Conclusion

The above study indicated clearly that the fungal strain *Alternaria alternata* AE1 exhibited excellent antimicrobial activity especially against both Gram positive and Gram negative bacteria and also showed cidal mode of action. The organism was able to produce a number of antimicrobial compounds in the extracellular broth. The extract of AE1 has exerted adverse effect on central carbohydrate metabolism of pathogenic bacteria. Besides, the EA extract of AE1 exhibited very strong free radical scavenging activity. Therefore the isolate can be considered as a prospective source of bioactive compounds for the development of new drugs.

## Supporting information

S1 Fig Zones of inhibition produced by CFS of AE1 grown in three different media against pathogenic bacteria: A- *B. subtilis*; B- *S. typhimurium*.
(TIF)
