# Supplementary figures and images for: Production of bioactive compounds with bactericidal and antioxidant potential by endophytic fungus Alternaria alternata AE1 isolated from Azadirachta indica A. Juss
Source: PLoS One. 2019 Apr 4;14(4):e0214744. doi: 10.1371/journal.pone.0214744 (PMC6448914; doi:10.1371/journal.pone.0214744)

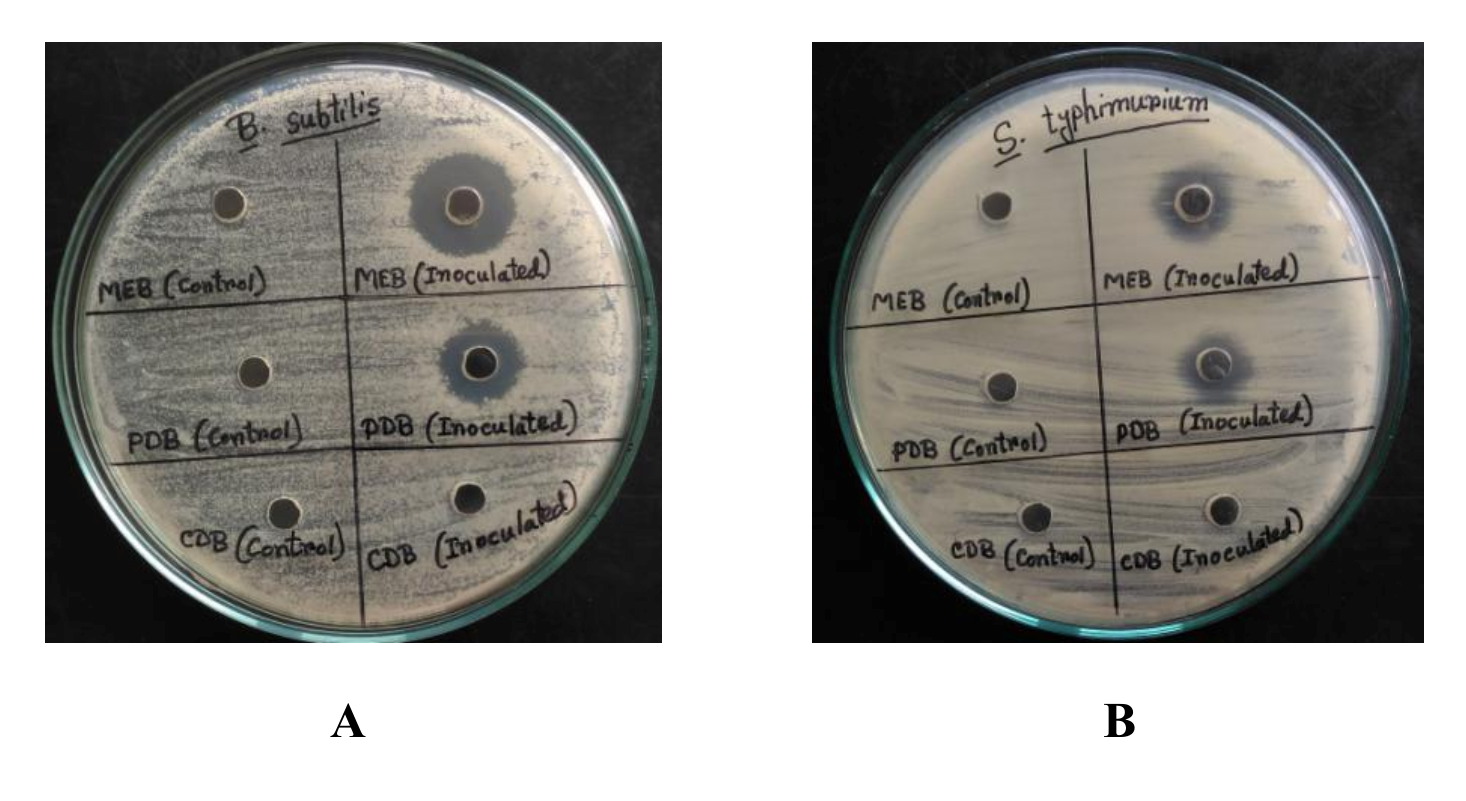

Supplement: S1 Fig — Zones of inhibition produced by CFS of AE1 grown in three different media against pathogenic bacteria: A- B. subtilis; B- S. typhimurium. (TIF) [file pone.0214744.s001.tif]
